# Comparison of Efficacy and Ocular Surface Assessment Between Preserved and Preservative-Free Brimonidine/Timolol Fixed-Combination Eye Drops in Glaucoma Patients: A Parallel-Grouped, Randomized Trial

**DOI:** 10.3390/jcm14051587

**Published:** 2025-02-26

**Authors:** Myungjin Kim, Chang-Kyu Lee, Jonghoon Shin, Doah Kim, Seungsoo Rho

**Affiliations:** 1 Department of Ophthalmology, CHA Bundang Medical Center, CHA University, Seongnam 13496, Republic of Korea; brandmjeyes@gmail.com (M.K.); inhamed93216006@gmail.com (D.K.); 2 Department of Ophthalmology, Ulsan University Hospital, University of Ulsan College of Medicine, Ulsan 44033, Republic of Korea; coolleo@uuh.ulsan.kr; 3 Department of Ophthalmology, Pusan National University Yangsan Hospital, Pusan National University School of Medicine, Yangsan 50612, Republic of Korea; jjongggal@naver.com; 4 Research Institute for Convergence of Biomedical Science and Technology, Pusan National University Yangsan Hospital, Yangsan 50612, Republic of Korea; 5 PNU Busan Eye Clinic, Busan 46241, Republic of Korea

**Keywords:** preservative-free, brimonidine/timolol, glaucoma, ocular hypertension, efficacy, safety, corneal staining score, conjunctival staining score, ocular surface disease index, drug tolerance, adherence

## Abstract

The objectives of the study were to compare the efficacy and safety using ocular surface assessment between preserved and preservative-free brimonidine/timolol fixed-combination eye drops in glaucoma or ocular hypertension patients. **Methods**: This study was designed as a prospective, multicenter (three institutions), investigator-masked, parallel-grouped randomized clinical trial. The primary outcomes were corneal and conjunctival staining score, ocular surface disease index (OSDI) score, drug tolerance, and adherence rates at 12-week visits. The secondary outcomes were corneal and conjunctival staining score, OSDI score at 4-week visits and intraocular pressure (IOP), tear-film break-up time (TBUT), and bulbar/limbal hyperemia score at the 4- and 12-week visits. For safety assessment, best-corrected visual acuity (BCVA), systolic blood pressure (SBP), diastolic blood pressure (DBP), heart rate (HR), and physical examination at 4 and 12 weeks and adverse events during the whole study period were analyzed. **Results**: Overall, 59 patients were enrolled and randomized into each group (29 preserved and 30 preservative-free). At the endpoint, 5 patients in the preserved group and 2 patients in the preservative-free group dropped out, leaving 24 and 28 patients in the preserved and preservative-free groups, respectively. Baseline characteristics showed no significant difference between the groups including age and sex. At the 12-week visit, intra-group change of OSDI scores did not change significantly compared to the baseline scores in both preserved and preservative-free groups (*p* = 0.791, 0.478, respectively). On the contrary, the corneal staining score and the conjunctival staining score showed a significant increase compared to the baseline score in the preserved group (*p* = 0.015, 0.009, respectively). Regarding drug satisfaction, higher proportions of patients in the preservative-free group reported convenience of installation (*p* = 0.002). Also, stinging and burning sensations in drug tolerance showed better results in the preservative-free group with a significant difference (*p* = 0.011). Safety assessment regarding systemic side effects such as SBP, DBP, and HR showed similar results between the preserved and preservative-free groups (*p* = 0.711, 0.232, 0.666, respectively). **Conclusions**: Preservative-free brimonidine/timolol showed comparable efficacy and safety, better corneal and conjunctival staining score with convenience of installation, and lower stinging and burning sensation. It is expected to be a proper treatment option for patients with glaucoma or ocular hypertension.

## 1. Introduction

Medication adherence is crucial for achieving optimal treatment outcomes regardless of a wide range of diseases. By identifying and addressing the drivers and barriers to adherence, physicians can better support their patients in adhering to their medication regimens, ultimately leading to improved treatment outcomes. However, a review spanning three decades found that between 30% and 50% of patients exhibit poor compliance with their prescribed treatments, regardless of disease type, prognosis, or care setting [1]. Glaucoma, which is characterized by chronic progressive damage to the optic nerve, is often asymptomatic until the advanced stage, making it more prone to poor patient adherence and persistence [2,3,4,5].

Previous studies have shown that factors such as younger age, male, poor general health status, and psychiatric conditions like depression negatively impact adherence to glaucoma medications [6,7,8]. In addition to these patient factors, the adherence rate may be dependent on the medication itself. As eyedrops contain not only active pharmaceutical ingredients but also excipients (non-active pharmaceutical ingredients) and preservatives, it is essential to thoroughly consider the potential impact of all these components when assessing their influence on adherence.

When administering eyedrops, several routes of absorption are possible and excessive amounts of active ingredients may cause unwanted systemic side effects. For example, brimonidine can lead to systemic adverse reactions, including dry mouth, dizziness, fatigue, and drowsiness, especially in predisposed bradycardic patients [9,10]. Eye drops with timolol also can have a strong and prolonged systemic effect such as decreased heart rate or cardiac output, and syncope especially in older age groups [10,11,12].

In addition to systemic effects, preservatives in the eye drops have been demonstrated to increase the patients’ discomfort associated with the therapy, contributing to the development of ocular surface disease (OSD) [13]. Benzalkonium chloride (BAK), a common preservative in ophthalmic solutions, is effective at preventing bacterial and fungal growth [14]. However, this cytotoxic property can also harm ocular surface cells, and studies indicate a correlation between the number of BAK-preserved drops used and the occurrence of adverse ocular surface signs and symptoms [15,16,17,18,19,20]. This association is further supported by observed improvements in ocular surface signs and symptoms followed by a discontinuation of the preserved drops and switch to preservative-free drops [18,21]. The occurrence of OSD in glaucoma patients is a considerable problem as it could be associated with reduced patients’ adherence to glaucoma eyedrop which leads to treatment failure [15,16,17,21,22,23,24]. Thus, the use of preservative-free eye drops becomes more popular, as they are likely to reduce or eliminate ocular side effects of preservatives.

Recently, a preservative-free brimonidine/timolol fixed-combination eyedrop has been developed in South Korea. The main purpose of this study was to prospectively compare the efficacy and safety of preservative-free brimonidine/timolol (Bridin plus^®^, Hanlim Pharm, Seoul, Republic of Korea) to preserved brimonidine/timolol (Combigan™, Allergan Inc., Irvine, CA, USA) in aspects of ocular surface assessment and adherence rates in patients with open-angle glaucoma or ocular hypertension. This study is a serial investigation following our prior research, which prospectively compared preserved and preservative-free latanoprost, and preserved and preservative-free brimonidine tartrate [25,26]. Moreover, to evaluate the potential benefits of preservative-free brimonidine/timolol, systemic parameters, questionnaire-based patient satisfaction, and drug tolerance scores were analyzed.

## 2. Materials and Methods

### 2.1. Study Design and Subject Enrollment

This prospective, multicenter (three institutions), investigator-masked, parallel-grouped randomized clinical trial aimed to assess the efficacy and safety of preserved and preservative-free brimonidine/timolol in patients with open-angle glaucoma or ocular hypertension. The study was approved by the institutional review boards of CHA Bundang Medical Center, Pusan National University Yangsan Hospital, and Ulsan University Hospital, adhered to the tenets of the Declaration of Helsinki, and was registered on clinicaltrials.gov on 8 October 2021 (NCT06078592). All subjects were enrolled from October 2021 to December 2022.

Glaucomatous changes in the eye were confirmed by consistent glaucomatous visual field defects that corresponded with characteristic optic disc and retinal nerve fiber layer abnormalities, as assessed by glaucoma specialists [27,28]. Each participant underwent a comprehensive ophthalmologic examination, which included best-corrected visual acuity (BCVA) measured using Snellen chart [29], intraocular pressure (IOP) assessment via Goldmann applanation tonometry by masked examiners in each institution [30,31], central corneal thickness (CCT) [30,32], gonioscopy [33,34], visual field test with a Humphrey Field Analyzer (Carl Zeiss Meditec, Dublin, CA, USA) [35], fundus photography (Carl Zeiss Meditec, Dublin, CA, USA) [36], red-free photography (Carl Zeiss Meditec, Dublin, CA, USA) [36], and spectral-domain optical coherence tomography (Carl Zeiss Meditec, Dublin, CA, USA) [37,38,39]. Additionally, baseline physical characteristics such as height and weight, were recorded using a standardized stadiometer and digital scale, with participants wearing light clothing and no shoes.

To be included in the study, patients needed to have an IOP between 15 and 40 mmHg in at least one eye during the screening visit, following the appropriate washout period. Exclusion criteria included BCVA worse than Snellen 20/80 (decimal 0.25), CCT outside the range of 470 to 591 μm, and any ocular condition (e.g., ischemic optic neuropathy, proliferative diabetic retinopathy, age-related macular degeneration) that could significantly affect visual field results. Patients with active ocular inflammatory conditions, a history of lacrimal punctal occlusion procedures within the past three months, or a need for eyedrops containing hyaluronic acid, cyclosporine, or diquafosol for severe dry eye disease were also excluded. Also, pregnant or currently nursing individuals were not eligible for participation.

All participants receiving IOP-lowering treatment underwent a washout period of four weeks, except for those using cholinergic eye drops and carbonic anhydrase inhibitors, who had a shorter washout period of five days. After the washout phase, they were randomly assigned to one of two groups: one receiving multi-dose preserved brimonidine/timolol (Combigan™, Allergan Inc., Irvine, CA, USA) and the other receiving unit-dose preservative-free brimonidine/timolol (Bridin plus^®^, Hanlim Pharm, Seoul, Republic of Korea).

**Figure 1:**
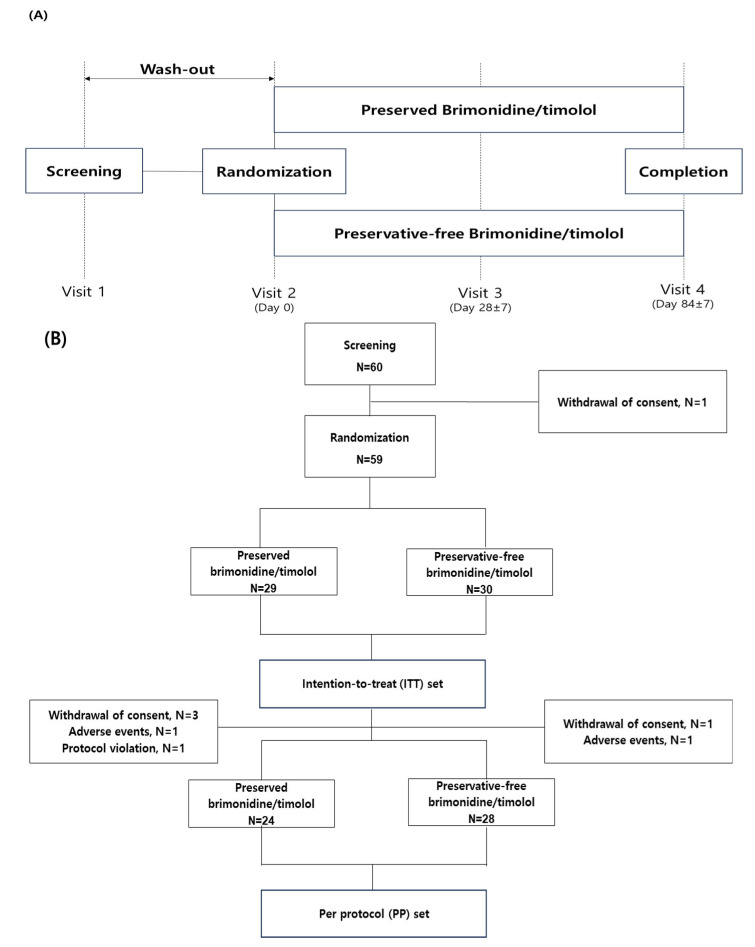
Flow chart of the study: (**A**) follow-up schedule; (**B**) subjects’ enrollment.

Randomization was conducted centrally and automatically using an interactive web-based randomization system (IWRS, TnW software Ltd., Seoul, Republic of Korea), which operated 24/7 throughout the study period. All patient data and study variables were recorded in a web-based electronic case report form (ver 1.0, http://www.ecrf.kr (accessed on 31 December 2022), TnW software Ltd., Seoul, Republic of Korea). To ensure masking, both treatment groups received their respective study medications in identical external packaging. While patients were aware of their assigned treatment, investigators remained blinded to the treatment allocation throughout the study period. The patients were instructed to instill either preserved or preservative-free brimonidine/timolol twice daily from day 0 and to visit the clinic at 4 and 12 weeks (Figure 1A). At both 4- and 12-week visits, the patients underwent follow-up measurements at 10 AM ± 1 h and instilled the eyedrops thereafter.

### 2.2. Outcome Measurements

The investigators performed evaluations at both the 4-week and 12-week visits (at 10 AM ± 1 h), ensuring consistency in capturing time-sensitive signs and symptoms. The primary endpoints were the difference in the corneal staining grade assessed by the Oxford grading system (0–5) and conjunctival staining score assessed by the National Eye Institute scale (0–3) [40,41] with FLUO 900 fluorescein strip (Haag-Streit, Köniz, Switzerland) staining under the slit lamp examination using a cobalt blue light. The ocular surface disease index (OSDI) score [42], drug tolerance, and adherence rates at 12-week visits were obtained. The drug tolerance data were acquired using a questionnaire sheet to evaluate the frequency and severity of the symptoms associated with using eye drops, including stinging/burning, sticky sensation, itching, blurring, sandiness/grittiness, dryness, light sensitivity, and pain/soreness. The level of each symptom was graded as 0 (none) to 3 (severe, immensely interfering with the subject’s daily life), and the duration of each symptom as 0 (prompt: <5 min) or 1 (continuous: ≥5 min). Adherence rates (0–100%) were assessed at 4 and 12 weeks using a self-report sheet [25,26].

The secondary efficacy endpoints were corneal and conjunctival staining score, and OSDI score at 4-week visits and IOP measured by Goldmann applanation, tear break-up time (TBUT) with FLUO 900 fluorescein strip staining under the slit lamp examination using a cobalt blue light, and bulbar and limbal hyperemia score assessed by Efron grading scale (0–4) [43] at the 4- and 12–week visits. For safety assessment, BCVA, systolic blood pressure (SBP), diastolic blood pressure (DBP), heart rate (HR) at 4 and 12 weeks, and adverse events (AEs) during the whole study period were analyzed.

### 2.3. Statistical Analysis

This study aimed to evaluate the superiority of preservative-free brimonidine/timolol over preserved brimonidine/timolol in terms of ocular surface. Superiority was concluded if the difference in the hyperemia score was 0.82 or more according to a study that evaluated the difference in eye redness before and after switching the subjects using preserved and preservative-free eyedrops [25,26,44,45]. Assuming a dropout rate of 25% and a standard deviation of 1.0 for the hyperemia score, a total of 60 patients (30 in each group) were required to achieve 90% power for the superiority calculation, resulting in a minimal sample size of 21 per group after accounting for dropouts [25,26,44,45]. To maximize the accuracy of the assessment among all investigators, a blinded person created a validation image set of conjunctival hyperemia, which was used to check the agreement between each investigator [25,26,44,45].

Baseline characteristics were compared between the preserved and preservative-free groups using independent *t*-tests or Wilcoxon rank-sum tests for continuous variables and chi-square or Fisher’s exact tests for categorical variables. Prior to selecting statistical tests, the normality of data distributions was assessed using the Shapiro–Wilk test. Variables that followed a normal distribution were analyzed using parametric tests, while non-parametric methods were used for non-normally distributed data. Inter-group comparisons of continuous outcome measurements at 4- and 12-week visits were performed using analysis of covariance (ANCOVA) after adjusting baseline values and covariates, if needed. Intra-group comparisons of serial measurements compared to baseline data were performed using paired *t*-test or Wilcoxon signed-rank tests. Assessments were performed using intention-to-treat (ITT) and per-protocol (PP) sets. Only patients who did not violate the protocol with an adherence rate of more than 80% were included in the PP set. Statistical analyses were performed using the Statistical Package for the Social Sciences for Windows (version 18.0; IBM Corp., Armonk, NY, USA), and statistical significance was set at *p* < 0.05.

## 3. Results

Overall, 59 patients were randomized into each group (29 preserved and 30 preservative-free group) which comprised an ITT set. During the study, five patients (three withdrawal of consent, one adverse event, one protocol violation) were excluded from the preserved group and two patients (one withdrawal of consent, one adverse event) from the preservative-free group. Consequently, 24 and 28 patients were included in the PP set of preserved and preservative-free groups, respectively (Figure 1B). There were no significant differences in the demographic and baseline features including age and sex, between the groups, minimizing potential confounding effects on variables such as BCVA, SBP, DBP, and HR (Table 1).

### 3.1. Primary Outcomes

**Figure 2:**
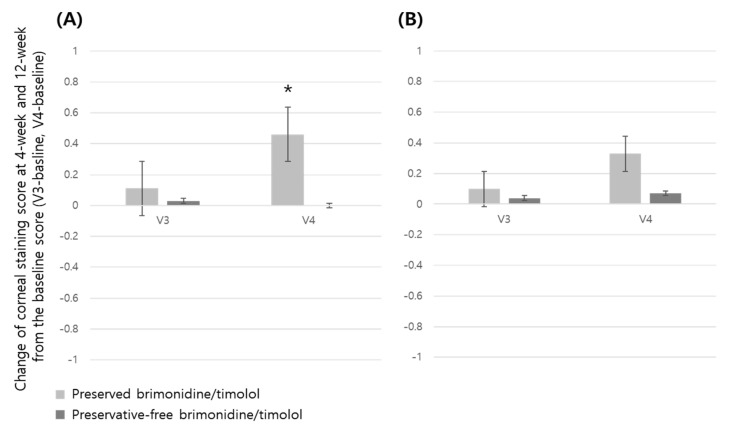
Intra-group change of corneal staining scores in preserved and preservative-free brimonidine/timolol groups at 4-week (V3) and 12-week (V4) visits from the baseline score. (* represents statistical significance by paired *t*-test). ITT, intention-to-treat set; PP, per-protocol set. (**A**) ITT, intention-to-treat set; (**B**) PP, per-protocol set.

**Figure 3:**
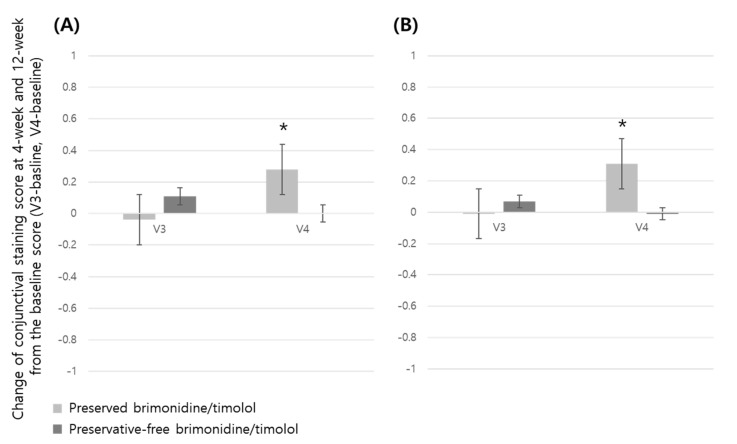
Intra-group change of conjunctival staining score in preserved and preservative-free brimonidine/timolol groups at 4-week (V3) and 12-week (V4) visits from the baseline score (* represents statistical significance by paired *t*-test). ITT, intention-to-treat set; PP, per-protocol set. (**A**) ITT, intention-to-treat set; (**B**) PP, per-protocol set.

The corneal and conjunctival staining scores, OSDI scores, drug tolerance, and patient satisfaction at 12-week visits were compared between the groups (Table 2). Although the corneal and conjunctival staining scores and OSDI scores did not differ significantly between the preserved and preservative-free groups at 12-week visits (Table 2), comparing intra-group changes of the corneal and conjunctival staining scores at 12-week visits from the baseline score for each group, preserved group showed increased corneal score in ITT set and increased conjunctival score in both the ITT and PP set, which means an increased ocular surface defect of cornea and conjunctiva in the preserved brimonidine/timolol group (Figure 2 and Figure 3).

Regarding drug tolerance, a stinging/burning sensation was more often associated with the preserved group in both ITT and PP sets with statistical significance (*p* = 0.011, 0.010, respectively; Table 2). Pain/soreness sensation was also higher in the preserved group in the PP set with statistical significance (*p* = 0.033; Table 2). In the patient satisfaction score, the preservative-free group reported that the unit-dose container was easier for installation in both ITT and PP set with statistical significance (*p* = 0.004, 0.002, respectively; Table 2), and easier for storage in the PP set with statistical significance (*p* = 0.030; Table 2).

**Figure 4:**
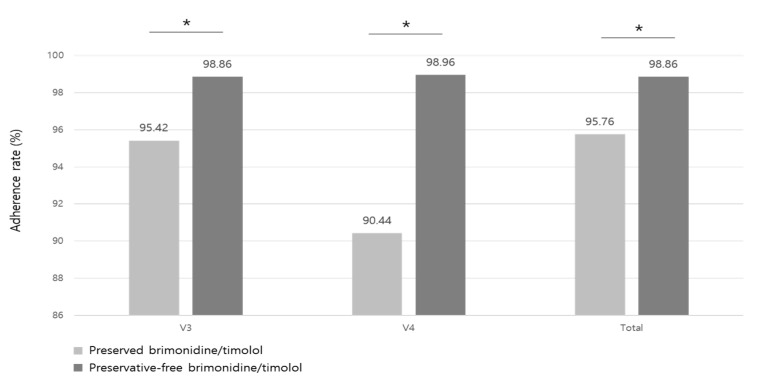
Adherence rate (%) between preserved and preservative-free brimonidine/timolol groups. (* represents statistical significance by independent *t*-test).

Adherence rates differed with statistical significance between the groups (Figure 4). At 4-week visits, the preserved group reported an adherence rate of 95.42%, while the preservative-free group reported 98.86% (*p* = 0.003). At 12-week visits, the adherence rate of the preserved group decreased to 90.44%, while the preservative-free group increased to 98.86% (*p* = 0.036).

### 3.2. Secondary Outcomes

**Figure 5:**
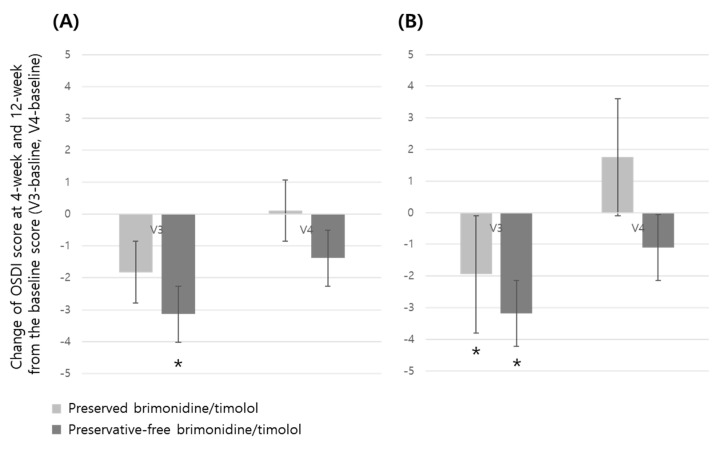
Intra-group change of OSDI score in preserved and preservative-free brimonidine/timolol groups at 4-week (V3) and 12-week (V4) visits from the baseline score. (* represents statistical significance by paired *t*-test). (**A**) ITT, intention-to-treat set; (**B**) PP, per-protocol set.

There were no statistically significant differences between the groups in corneal and conjunctival staining score and OSDI score at 4-week visits. Also, the TBUT score and bulbar/limbal hyperemic score did not differ significantly between the groups at 4-week and 12-week (Table 3) visits. However, intra-group differences of the OSDI score were observed in the preservative-free group at 4-week visits in the ITT set, and in both preserved and preservative-free groups at 4-week visits in the PP set (Figure 5). In addition, although both groups showed decreased OSDI scores compared with the baseline at 4-week visits, the OSDI score change showed a different tendency at 12-week visits in both groups. The preserved group showed increased OSDI scores compared with the baseline at 12-week visits, while the preservative-free group showed consistently decreased OSDI scores (Figure 5). Other variables including bulbar and limbal hyperemic score and IOP at 4 and 12 weeks showed no statistically significant differences between the groups. Both groups showed significantly lower IOPs at both 4 and 12 weeks compared to those at baseline, with no significant differences between the groups throughout the study period.

### 3.3. Safety Assessments

The BCVA, SBP, DBP, and HR did not differ between the groups throughout the study period, with all variables in the normal range (Table 4). The total incidence of AEs was 25.42% (15/59 patients, 16 cases), which was 24.14% (7/29 patients, 8 cases) in the preserved group and 26.67% (8/30 patients, 8 cases) in the preservative-free group. The AEs that caused the drop-out in the study were palpitations in the preserved group and dizziness in the preservative-free group. However, severe AEs were not reported, and all participants recovered to a normal state after discontinuing the eyedrops.

## 4. Discussion

Ocular surface can be affected not only by preservatives like BAK, but also by active pharmaceutical ingredients or other excipients with which BAK is combined. Several in vitro and ex vivo studies have shown that BAK alone, at concentrations commonly found in commercial formulation, and BAK combined with timolol, have more deleterious effects on conjunctival cells compared to BAK used with prostaglandin analogues [46,47,48,49]. Similarly, the fact that conjunctiva staining score showed differences between the groups at 4 weeks in our study implies that the active pharmaceutical ingredient should also be under consideration for the cause of OSD. Sherwood et al. [50] demonstrated that in the 12-month clinical trial, the most frequent treatment-related AEs by brimonidine/timolol recipients (n = 385) were conjunctival hyperemia (14.5%), followed by ocular stinging (6.2%), eye pruritus (5.5%), and allergic conjunctivitis (5.2%). Interestingly, ocular stinging was more frequent with brimonidine/timolol therapy than with brimonidine alone (*p* = 0.03), though other adverse events were less common with brimonidine/timolol (*p* < 0.02). These results support the fact that the impact on the ocular surface or ocular side effects can vary depending on the composition of active ingredients as well. On the contrary, our results showed that both bulbar and limbal hyperemic scores rather decreased at 12 weeks compared to baseline scores in both preserved and preservative-free brimonidine/timolol groups, although they did not show statistical differences. The impact of brimonidine/timolol on hyperemia seems to manifest between 3-month and 12-month intervals, highlighting the need for increased attention to conjunctival hyperemia during this period which may be due to allergic reaction. We also evaluated stinging/burning sensation to assess drug tolerance. There were statistically significant differences in the stinging/burning sensation between the preserved and preservative-free groups in both ITT and PP sets. Thus, starting with preservative-free brimonidine/timolol could be beneficial to improve ocular comfort and tolerance from the outset, avoiding potential issues related to hyperemia and ocular discomfort. Also, switching from preserved brimonidine/timolol to preservative-free brimonidine/timolol would be another treatment option for patients experiencing ocular a stinging sensation.

Even with topical administration, brimonidine and timolol can enter systemic circulation, potentially causing cardiovascular (brimonidine, timolol) and pulmonary (timolol) effects [51,52,53,54]. Stewart et al. [54], reported that the use of concomitant timolol maleate and brimonidine, given as separate agents, was related with ventricular premature contractions during exercise, and atrial premature contractions during recovery in treatment groups. On the contrary, in our study in which brimonidine and timolol were given in a fixed-combination formula, there were no significant systemic AEs in the safety assessment. The components used in formulating fixed combinations of brimonidine and timolol appear to possess the potential to mitigate these cardiovascular and pulmonary effects associated with each individual use of brimonidine or timolol. Meanwhile, our previous study [26] reported lower SBP and DBP at 4 and 12 weeks compared to baseline measures when using preserved brimonidine, but not in the preservative-free brimonidine group. The important factor could be the extent of individual drug consumption. Since bottle-type dispensers are not transparent, patients may have inadvertently instilled multiple drops at one session, resulting in overconsumption. In contrast, no such association was identified for unit-dose pipettes [55]. Furthermore, the difference in total amount of brimonidine derived from the different frequency of use and the concentration would have influenced the results. In our previous study [26], the brimonidine was required to be instilled three times a day, whereas in the current study, brimonidine/timolol was required to be instilled twice daily. Brimonidin (Alphagan P^®^, Allergan Inc., Irvine, CA, USA) is typically prescribed as 0.15% administered three times daily, totaling 0.45% per day, whereas brimonidine/timolol (Combigan™, Allergan Inc., Irvine, CA, USA) is usually prescribed as 0.2% administered twice daily, totaling 0.4% per day.

Along with several previous studies demonstrating that chronic use of preserved eye drops causes significant damage to the ocular surface [13,17,21,24,25,26,56,57,58], we found that intra-group changes of the corneal and conjunctival staining scores at 12-week visits from the baseline score for each group showed different tendencies. The preserved group showed an increased corneal score in the ITT set and increased conjunctival score in both ITT and PP sets, which means an increased ocular surface defect of cornea and conjunctiva in the preserved brimonidine/timolol group. Also, we found that the TBUT was improved in the preservative-free group compared to preserved group, and the preserved group showed an increased OSDI score compared with the baseline at 12 weeks, while the preservative-free group showed consistently a decreased OSDI score which means better ocular surface status. These results imply that preservative-free brimonidine/timolol may reduce adverse effects of BAK on ocular surface. The higher patient adherence rate in the preservative-free group may be attributed to reduced BAK-related adverse effects, supported by the prior findings showing self-reported nonadherence rates of 32.0% for the preserved eye drops and 12.5% for the preservative-free eye drops [3].

Although inter-group change of the corneal and conjunctival staining and OSDI scores did not differ significantly between the preservative-free and preserved groups at 12-week visits, it does not mean the minimized adverse effect of BAK. A previous study comparing BAK-preserved latanoprost to BAK-free travoprost found that in the overall cohort of patients, mean OSDI scores at the 12-week time point were not statistically different, but significant improvement was seen in the subsets of patients with mild OSDI scores at baseline and patients who had long-term (more than 24 months) exposure to BAK-preserved latanoprost before entry into the study [24]. The latter finding suggests a cumulative effect of BAK, consistent with its persistence in ocular tissues [13]. On the contrary, the acute detrimental effect of BAK does not last long if the exposure time is a fairly short term. A recent study described that preserved latanoprost caused an acute decrease in the corneal epithelium at 1 min after the first instillation [56]. However, the decrease disappeared at 24 h after a once-daily application of the preserved latanoprost, which was also verified by scanning electron microscopy analyses [56]. This regenerative power in response to daily exposures to BAK may explain the lack of significant differences between the groups in ocular surface findings and OSDI scores during 4- and 12-week follow-up periods in this study. Alternatively, chronic changes from cumulative BAK toxicity might have been pronounced, potentially revealing a more significant detrimental effect on the corneal surface, if the follow-up period were longer [59]. On the other hands, intra-group changes of the corneal and conjunctival staining scores showed differences between the groups. In the preserved group, the corneal staining score continuously increased at 4 and 12 weeks compared to the baseline score, but in the preservative-free group, the scores were similar or showed decreasing tendency compared to the baseline score. This could possibly be related to the timing of the loss of corneal regenerative power due to the accumulation of BAK in the twice-daily use. Additionally, concerning the conjunctival staining score, there was a different trend between the groups. The preservative-free group showed no change at 4 and 12 weeks, while the preserved group showed an increased score at 12 weeks.

Ensuring patient adherence is a major concern for glaucoma treatment, as it significantly impacts patients’ life-long medication use and influences the overall disease progression. In our study, adherence rates were consistently higher in the preservative-free group compared to the preserved group throughout the study period. Key differences between the two groups were differences in stinging/burning sensation and pain/soreness scores of drug tolerance, and easiness for instillation and storage of patient satisfaction scores. Participants using preservative-free brimonidine/timolol reported a lower stinging/burning sensation or pain/soreness score which could be related to ingredients in eye drop itself as mentioned above. Higher levels of patient satisfaction suggest that the single-dose container was both easy to use and convenient for storage. Although there is ongoing debate about whether elderly patients may struggle with opening rigid plastic containers of singe-dose units [60,61], this issue was not considered a significant hurdle for the newly developed preservative-free formulation. Moreover, patients can easily count the number of used or unused doses with the single-dose container system or keep the drug in desired locations, leading to better drug accessibility, resulting in convenient drug storage, another advantage of the preservative-free formulation in this study. Similar results were also reported in the previous study [26].

Since glaucoma is a chronic progressive disease property, long-term use of a glaucoma agent is often required. Initial treatment typically involves a single topical agent (monotherapy), but additional agents (combination therapy) are frequently needed to reach the target IOP [62,63,64]. Meanwhile, modern adjunctive therapy combines a β-receptor antagonists with another class of drug [65], and one of them is fixed-combination brimonidine/timolol which consists of brimonidine 0.2% and timolol 0.5%. In the Ocular Hypertension Treatment Study, 40% of treated subjects required more than one medication to achieve the therapeutic goal of 20% IOP reduction from baseline [62,64]. More than 75% of subjects in the medical treatment arm of the Collaborative Initial Glaucoma Treatment Study required at least two medications after 2 years [63]. Unfortunately, anti-glaucomatous eye drops often have significant adverse effects including OSD, which when combined with the silent nature of glaucoma leads to a high risk of low adherence if patients do not understand their disease and the importance of treatment [66]. Erb et al. [67] reported that the prevalence of dry eye disease in glaucoma was 52.6% and increased with age, the duration of glaucoma, and the number of eye drops used (1 eye drop 50.9%, 5 eye drops 66.7%). Therefore, use of fixed-combined drugs as treatment strategy to reduce the number of ophthalmic solutions may be reasonable while maintaining the comparable IOP-lowering effect and enhancing patient adherence.

This study has several limitations that should be considered when interpreting the findings. First, the relatively short follow-up period of 12 weeks may not fully capture the long-term effects of preservative-free versus preserved brimonidine/timolol on ocular surface health. Extended observation periods could provide a more comprehensive understanding of the chronic impacts on the ocular surface and adherence. Second, the dropout rate, although accounted for in the power calculation, led to a slightly uneven sample size between the groups, which may affect the statistical power in subgroup analyses. Additionally, the study was conducted across three institutions in South Korea, which may limit the generalizability of the results to populations with different demographic or environmental characteristics. Lastly, objective measures of inflammation, such as MMP-9 levels, were not included. Future studies incorporating biomarkers for ocular surface inflammation may yield further insights into the differential effects of preserved and preservative-free formulations.

In sum, subjects with preservative-free brimonidine/timolol fixed-combination eyedrops showed better ocular surface status and higher patient adherence compared to the preserved formulation, especially in corneal/conjunctival staining scores and OSDI scores. Through serial investigations comparing patients’ ocular surface status and adherence rate between the preserved and preservative-free eye drops [25,26], this study clearly demonstrates superiority of preservative-free eye drops on glaucoma patients in clinical application. Additionally, we discussed the influence of active pharmaceutical ingredients on ocular surface health, emphasizing the importance of considering both preservatives and active ingredients. Overall, our findings suggest that starting with preservative-free brimonidine/timolol rather than preserved formulation could be beneficial to improve the glaucoma drug adherence. Further studies with patients having different corneal properties and longer follow-up may be required to investigate the long-term effect of preservative-free and preserved medications in diverse settings.

## Figures and Tables

**Table 1:** Demographic and baseline characteristics between the preserved and preservative-free brimonidine/timolol groups (ITT set). *SBP*, systolic blood pressure; *DBP*, diastolic blood pressure; *HR*, heart rate; *BCVA*, best-corrected visual acuity; *IOP*, intraocular pressure; *CCT*, central corneal thickness; *OSDI*, ocular surface disease index.

	Preserved (n = 29)	Preservative-Free (n = 30)	*p*
Age, years	59.21 ± 11.47	57.27 ± 12.42	0.480
Male, n (%)	18 (62.1)	18 (60.0)	0.871
Duration of disease, years	0.45 ± 2.17	1.19 ± 3.52	0.834
Height, cm	166.07 ± 8.21	165.12 ± 7.33	0.640
Weight, kg	67.43 ± 10.89	67.23 ± 10.34	0.945
SBP, mmHg	129.21 ± 12.68	131.83 ± 14.01	0.454
DBP, mmHg	76.72 ± 9.34	78.10 ± 10.51	0.597
HR, beat per minute	75.69 ± 9.01	78.57 ± 9.08	0.249
BCVA (decimal)	0.88 ± 0.26	0.92 ± 0.22	0.512
IOP, mmHg	19.21 ± 2.84	18.71 ± 3.31	0.309
CCT, μm	546.76 ± 34.20	544.03 ± 27.24	0.499
Bulbar hyperemia	1.11 ± 0.69	0.83 ± 0.71	0.136
Limbal hyperemia	0.86 ± 0.71	0.79 ± 0.68	0.746
Corneal staining score	0.57 ± 0.69	0.79 ± 0.68	0.192
Conjunctival staining score	0.68 ± 0.62	0.90 ± 0.61	0.152
Tear film break up time	7.50 ± 6.26	7.69 ± 6.36	0.924
OSDI score	7.86 ± 6.41	7.37 ± 6.10	0.789

**Table 2:** Primary outcome measurements at 12-week visit. *OSDI* ocular surface disease index. ^a^ Statistical analyses were conducted using ANCOVA after adjusting baseline data. ^b^ Statistical analyses were conducted using independent *t*-test or Wilcoxon’s rank sum test. Significant values with *p* < 0.05 are indicated in bold.

	Intention-to-Treat Set			Per-Protocol Set		
	Preserved (n = 29)	Preservative-Free (n = 30)	*p*	Preserved (n = 24)	Preservative-Free (n = 28)	*p*
Corneal staining score (V4)	1.08 ± 0.15	0.75 ± 0.15	0.125 ^a^	0.91 ± 0.16	0.81 ± 0.14	0.630 ^a^
Corneal staining score difference from baseline (V4-baseline)	0.46 ± 0.88	0.00 ± 0.89	0.060	0.33 ± 0.80	0.07 ± 0.87	0.310 ^b^
Conjunctival staining score (V4)	1.02 ± 0.09	0.85 ± 0.09	0.211 ^a^	1.00 ± 0.11	0.84 ± 0.10	0.325 ^a^
Conjunctival staining score difference from baseline (V4-baseline)	0.28 ± 0.53	0.00 ± 0.62	0.073	0.31 ± 0.57	−0.01 ± 0.64	0.084 ^b^
OSDI score (V4)	7.65 ± 1.36	6.27 ± 1.34	0.473 ^a^	9.49 ± 1.62	6.36 ± 1.43	0.153 ^a^
OSDI score difference from baseline (V4-baseline)	0.11 ± 7.92	−1.38 ± 8.02	0.835	1.76 ± 8.09	−1.11 ± 8.02	0.423 ^b^
Drug tolerance score						
Stinging/burning	0.82 ± 0.67	0.38 ± 0.49	**0.011**	0.86 ± 0.65	0.37 ± 0.49	**0.010 ^b^**
Sticky	0.29 ± 0.53	0.10 ± 0.41	0.076	0.29 ± 0.46	0.11 ± 0.42	0.070 ^b^
Itching	0.29 ± 0.53	0.28 ± 0.65	0.727	0.29 ± 0.56	0.30 ± 0.67	0.910 ^b^
Blurred vision	0.54 ± 0.64	0.38 ± 0.56	0.341	0.62 ± 0.67	0.37 ± 0.56	0.173 ^b^
Sandiness/grittiness	0.36 ± 0.56	0.17 ± 0.38	0.182	0.38 ± 0.50	0.19 ± 0.40	0.138 ^b^
Dryness	0.43 ± 0.74	0.28 ± 0.45	0.603	0.48 ± 0.75	0.30 ± 0.47	0.486 ^b^
Light sensitivity	0.17 ± 0.47	0.43 ± 0.69	0.100	0.19 ± 0.48	0.52 ± 0.75	0.064 ^b^
Pain/soreness	0.36 ± 0.49	0.17 ± 0.38	0.120	0.43 ± 0.51	0.15 ± 0.36	**0.033 ^b^**
Patient satisfaction score						
Easy to open	1.61 ± 0.74	1.31 ± 0.54	0.109	1.71 ± 0.78	1.33 ± 0.55	0.075 ^b^
Easy for installation	1.89 ± 0.74	1.38 ± 0.68	**0.004**	2.05 ± 0.74	1.41 ± 0.69	**0.002 ^b^**
Easy for storage	1.61 ± 0.69	1.38 ± 0.62	0.163	1.81 ± 0.68	1.41 ± 0.64	**0.030 ^b^**
Easy for drug management	1.39 ± 0.74	1.17 ± 0.47	0.251	1.52 ± 0.81	1.19 ± 0.48	0.110 ^b^

**Table 3:** Secondary outcome measurements at 4-week visits (V3) and 12-week visits (V4). *OSDI,* ocular surface disease index; *TBUT,* tear-film break up time; *IOP,* intraocular pressure. ^a^ Statistical analyses were conducted using ANCOVA after adjusting baseline data. ^b^ Statistical analyses were conducted using independent *t*-test or Wilcoxon’s rank sum test.

	Intention-to-Treat Set			Per-Protocol Set		
	Preserved (n = 29)	Preservative-Free (n = 30)	*p*	Preserved (n = 24)	Preservative-Free (n = 28)	*p*
Corneal staining score (V3)	0.72 ± 0.13	0.79 ± 0.13	0.702	0.66 ± 0.16	0.78 ± 0.14	0.560 ^a^
Corneal staining score difference from baseline (V3-baseline)	0.11 ± 0.83	0.03 ± 0.78	0.619	0.10 ± 0.94	0.04 ± 0.81	0.722 ^b^
Conjunctival staining score (V3)	0.71 ± 0.12	0.95 ± 0.12	0.162	0.69 ± 0.14	0.91 ± 0.13	0.264 ^a^
Conjunctival staining score difference from baseline (V3-baseline)	−0.04 ± 0.57	0.11 ± 0.77	0.735	−0.01 ± 0.60	0.07 ± 0.76	0.478 ^b^
OSDI score (V3)	5.72 ± 0.82	4.52 ± 0.80	0.298	5.81 ± 0.97	4.26 ± 0.85	0.236 ^a^
OSDI score difference from baseline (V3-baseline)	−1.82 ± 4.36	−3.14 ± 6.89	0.195	−1.95 ± 4.01	−3.19 ± 7.11	0.452 ^b^
TBUT (V3), sec	6.34 ± 0.44	6.55 ± 0.43	0.728	6.24 ± 0.53	6.52 ± 0.47	0.699 ^a^
TBUT difference from baseline (V3-baseline)	−0.16 ± 2.57	0.22 ± 2.62	0.892	−0.33 ± 2.90	0.09 ± 2.61	0.604 ^b^
TBUT (V4), sec	6.43 ± 0.46	6.69 ± 0.46	0.689	6.54 ± 0.57	6.75 ± 0.51	0.781 ^a^
TBUT difference from baseline (V4-baseline)	−0.08 ± 3.05	0.36 ± 2.38	0.550	−0.03 ± 3.30	0.31 ± 2.45	0.683 ^b^
Hyperemic score						
Bulbar (V3)	0.55 ± 0.10	0.64 ± 0.10	0.510	0.38 ± 0.11	0.63 ± 0.10	0.101 ^a^
Bulbar hyperemic score difference from baseline (V3-baseline)	−0.50 ± 0.69	−0.24 ± 0.64	0.281	−0.67 ± 0.76	−0.26 ± 0.66	0.094 ^b^
Bulbar (V4)	0.70 ± 0.10	0.64 ± 0.10	0.659	0.62 ± 0.12	0.63 ± 0.10	0.982 ^a^
Bulbar hyperemic score difference from baseline (V4-baseline)	−0.36 ± 0.68	−0.24 ± 0.69	0.774	−0.43 ± 0.75	−0.26 ± 0.71	0.632 ^b^
Limbal (V3)	0.56 ± 0.11	0.60 ± 0.11	0.818	0.38 ± 0.13	0.59 ± 0.11	0.221 ^a^
Limbal hyperemic score difference from baseline (V3-baseline)	−0.29 ± 0.66	−0.21 ± 0.82	0.956	−0.43 ± 0.68	−0.22 ± 0.85	0.556 ^b^
Limbal (V4)	0.81 ± 0.10	0.70 ± 0.10	0.467	0.76 ± 0.13	0.70 ± 0.11	0.721 ^a^
Limbal hyperemic score difference from baseline (V4-baseline)	−0.04 ± 0.51	−0.10 ± 0.82	0.507	−0.05 ± 0.59	−0.11 ± 0.80	0.593 ^b^
IOP (V3), mmHg	13.89 ± 0.43	13.32 ± 0.42	0.348	13.16 ± 0.38	13.19 ± 0.33	0.949 ^a^
IOP difference from baseline (V3-baseline)	−5.20 ± 2.92	−5.52 ± 2.52	0.785	−6.24 ± 2.19	−5.78 ± 2.35	0.186 ^b^
IOP (V4), mmHg	13.71 ± 2.80	12.34 ± 2.45	0.054	13.27 ± 0.48	12.57 ± 0.42	0.280 ^a^
IOP difference from baseline (V4-baseline)	−5.50 ± 2.94	−6.36 ± 2.78	0.260	−6.14 ± 2.51	−6.39 ± 2.83	0.755 ^b^

**Table 4:** Safety assessment at 4- and 12-week visits using per-protocol set. *BCVA,* best-corrected visual acuity; *SBP,* systolic blood pressure; *DBP,* diastolic blood pressure; *HR,* heart rate. Comparative analyses were performed using ANCOVA after adjusting age, gender, and baseline data, except for BCVA. For BCVA, the Wilcoxon rank-sum test was used.

	4-Week Visit			12-Week Visit		
	Preserved (n = 29)	Preservative-Free (n = 30)	*p*	Preserved (n = 24)	Preservative-Free (n = 28)	*p*
BCVA (decimal)	0.90 ± 0.24	0.94 ± 0.25	0.438	0.88 ± 0.24	0.96 ± 0.24	0.145
SBP, mmHg	127.58 ± 9.23	128.39 ± 13.58	0.806	126.18 ± 11.25	128.00 ± 8.02	0.313
SBP-baseline, mmHg	−1.50 ± 10.88	−2.89 ± 14.04	0.6947	−2.71 ± 11.26	−3.90 ± 12.60	0.7105
DBP, mmHg	76.72 ± 9.34	78.10 ± 10.51	0.598	71.46 ± 6.06	72.34 ± 5.30	0.249
DBP-baseline, mmHg	1.75 ± 9.52	−1.18 ± 9.71	0.2792	1.46 ± 10.96	−1.83 ± 9.59	0.2322
HR, bpm	73.29 ± 7.84	72.07 ± 9.25	0.406	72.34 ± 5.30	71.46 ± 6.06	0.453

## Data Availability

The data presented in this study are available on request from the corresponding author. The data are not publicly available due to privacy.
